# Enolase and Acute Spinal Cord Injury

**DOI:** 10.4172/2155-9899.1000536

**Published:** 2017-12-29

**Authors:** Rachel Polcyn, Mollie Capone, Azim Hossain, Denise Matzelle, Naren L. Banik, Azizul Haque

**Affiliations:** 1 Department of Microbiology and Immunology, Hollings Cancer Center, Medical University of South Carolina, Charleston, USA; 2 Department of Neurosurgery, Medical University of South Carolina, Charleston, USA; 3 Department of Immunology, Ralph H. Johnson Veterans Administration Medical Center, Charleston, SC, USA

## Commentary

Spinal cord injury (SCI) is a debilitating neurological condition that affects approximately 285,000 people living in the United States [1]. Each year there are 17,500 new cases of SCI, most of whom are males (81%). Less than 1% of people hospitalized for this injury recover complete neurological function by the time they are discharged, and for most, the effects are life-long. Depending on age of incidence and extent of injury, the estimated lifetime cost for health care and living expenses following SCI can be as high as 4 million dollars. Despite the significant lifestyle and financial impact of this condition, no FDAapproved pharmaceutical treatment is available for SCI [2,3]. This commentary discusses enolase as a contributor to secondary mechanisms of acute SCI and a novel potential therapeutic, ENOblock, for attenuating these damages.

SCI typically occurs in two stages: primary and secondary [2,3]. The primary injury is the mechanical damage to the spinal cord from laceration, contusion, compression, and contraction of the tissue during the initial insult. Following the primary injury, additional damage to the neural tissue occurs from secondary injury, such as hypoxia/ischemia, glutamate excitotoxicity, inflammation leading to neuronal cell death, Wallerian degeneration, and glial scarring. Though primary injury damages are irreversible, several potential therapeutic targets lie within the molecular cascades involved in secondary injury, which begins minutes, hours, and days after the initial injury and provides a therapeutic window for treatment of partially damaged cells and tissue for improvement of function. Traditionally, methylprednisolone (MP) has been prescribed to protect against secondary injury damages from acute SCI. However, the increased risk of infection and myopathy with use of this treatment and controversy over whether the drug’s marginal benefits are sufficient to offset its risks, have prevented the therapeutic from garnering FDA-approval [2,3]. As of 2013, the official stance of the American Association of Neurological Surgeons and Congress of Neurological Surgeons is that use of MP in treatment of acute SCI is not recommended [4]. Other potential treatments targeting secondary injury mechanisms (i.e. Nimodipine, Gacyclidine, Thyrotropin Releasing Hormone, GM1 Ganglioside, Rho Antagonist (Cethrin), Anti-Nogo Antibodies, Acidic Fibroblast Growth Factor, Minocycline, Oxycyte, Premarin, and Riluzole) have been investigated, but FDA-approval remains elusive [2,3]. Thus, continued exploration of alternative pharmaceutical therapeutics and therapeutic targets will be vital for improving quality of life of affected individuals.

**Figure 1:**
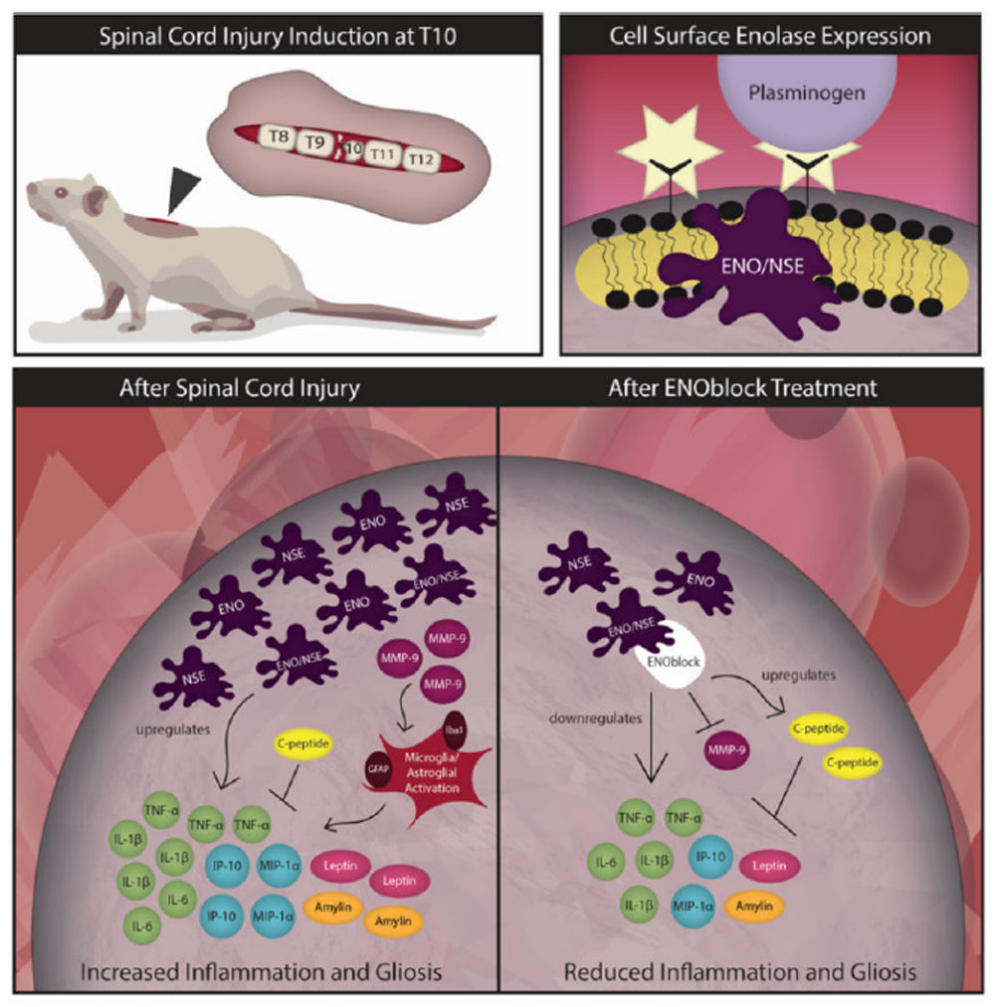
Changes associated with enolase/NSE expression, activity, and inhibition following acute spinal cord injury.

Under normal conditions, enolase, a multifunctional enzyme abundantly expressed in the cytosol, functions in glucose metabolism by catalyzing the conversion of 2-phosphoglycerate to phosphoenolpyruvate [5-8]. However, an accumulation of data implicating enolase as a neurotrophic factor with roles in hypoxia and ischemia, secondary factors contributing to neurodegeneration, led to an investigation of the enzyme’s involvement in SCI conditions [9]. Upon inflammatory signal, enolase can migrate from the cytosol to the cell surface where it enhances antigen presentation for host cell invasion *via* plasmin activation (Figure 1) and subsequent extracellular matrix degradation [7]. This cell surface expression of enolase can also trigger the production of reactive oxygen species (ROS), nitric oxide (NO), and pro-inflammatory cytokines (TNF-α, IL-1β, IFN-γ, and TGF-β) and chemokines (MCP-1 and MIP-1α) to bolster neurodegenerative response [7,10]. Upregulation of enolase levels have been reported following injury in a rat model, indicating this enzyme’s potential involvement in neurodegeneration or neuronal survival mechanisms related to SCI [9].

There are three distinct, tissue-specific isoforms of enolase: α-enolase (non-neuronal enolase, ENO1), γ-enolase (neuron specific enolase, NSE or ENO_2_), and β-enolase (muscle specific enolase, ENO3) [7]. During injury, α-enolase, mostly found in adult tissues, is converted to NSE in neurons and cells of neuroendocrine origin and to β-enolase in muscle. NSE isoforms have also been found in microglia, oligodendrocytes, and astrocytes, indicating a connection between NSE expression and glial cell function [7,8]. NSE is considered a biomarker of functional damages to neurons in SCI because of its unique location in neural tissues and upregulated secretion following axonal damage [6,7]. Most recently, the effects of altering enolase/NSE expression and activity following SCI were explored for the first time to further clarify the enzyme’s functions in neurodegeneration associated with secondary injury mechanisms of SCI [6].

ENOblock (C31H43FN8O3), a novel small molecule inhibitor of enolase, was administered to male Sprague-Dawley rats at 15 min and 24 h post-injury induction at T10 to test the effects of the enzyme on SCI mechanisms. Haque et al. reported increased NSE expression and activity in glial and neuronal cells following acute SCI, and they showed that this increased expression was attenuated with ENOblock treatment. In addition, enolase inhibition with ENOblock corresponded to a reduction in inflammatory chemokines and cytokines, key mediators of secondary injury damages associated with SCI. Expression of pro-inflammatory cytokines (TNF-α, IL-1β, and IL-6) during secondary SCI can induce hyperalgesia, allodynia, and apoptosis of neuronal and glial cells [6,11-14]. Additionally, the chemokine IP-10 is expressed by astrocytes in response to NMDA-dependent excitotoxicity and has been implicated in mediating cell proliferation and apoptosis under neurodegenerative conditions *via* MAPK/ERK signaling pathways [15,16]. Recently, increased serum expression of inflammatory cytokines (TNF-α, IL-1β, and IL-6) and chemokines (MIP-1α and IP-10) was found in response to SCI, which was markedly decreased within 2 days of ENOblock treatment [6]. Thus, the attenuation of inflammatory chemokines and cytokines with ENOblock could be a useful therapeutic strategy to reduce neuronal cell death and pain response following acute SCI.

Further analysis of the metabolic factors influenced by enolase/NSE inhibition with ENOblock, namely C-peptide, leptin, and amylin, has shown a connection between reduced enolase activity and anti-inflammatory response [6]. C-peptide, known for having anti-inflammatory activity, is significantly elevated following ENOblock treatment. This change is supportive of favorably modulating inflammation towards neuronal survival after the injury occurs. Additionally, leptin, a hormone involved in chronic inflammation pathology, and amylin, a peptide hormone associated with inflammation in a variety of injuries, have been shown to be significantly elevated following SCI. ENOblock treatment has reduced leptin and amylin levels in acute SCI rats. These results indicate that ENOblock inhibition of enolase/NSE may be a potential therapeutic strategy for favorably modulating metabolic response to SCI towards neuroprotection and should be further investigated.

In addition to attenuation of inflammatory chemokines/cytokines and modulation of metabolic factors, enolase inhibition with ENOblock has been shown to influence microglial and astroglial activation to reduce gliosis [6]. Elevated MMP-9 expression promotes microglia and astrocyte activation, leading to inflammatory chemokine and cytokine release that promotes cell death. The observed increase in MMP-9 expression following SCI and subsequent decrease with ENOblock treatment indicate a role for enolase inhibition in modulating glial cell activation of inflammatory cascades following acute SCI. These changes reflect those observed for NSE expression and activity, indicating the potential for an association between the two mechanisms that could be further elucidated in future studies. ENOblock treatment also decreases expression of Iba1, a microglial marker that is upregulated following SCI, and GFAP, an astroglial marker increased following SCI, in injured tissues. Based on this information, blocking inflammatory cascades by inhibiting MMP-9 with ENOblock could be an effective treatment strategy for reducing SCI secondary damages from inflammation. ENOblock inhibition of enolase/NSE may offer additional therapeutic effects by attenuating gliosis following acute SCI.

The first evaluation of ENOblock inhibition of enolase/NSE expression and activity in a rat model of acute SCI has revealed several promising indicators (Figure 1) of this treatment’s favorable influence over inflammatory events leading to secondary damages [6]. NSE serum and tissue levels are markedly elevated following acute SCI and can be attenuated with the enolase inhibitor, ENOblock. Furthermore, ENOblock treatment has been shown to decrease inflammatory chemokines/cytokines, inhibit MMP-9 activation, reduce gliosis, and modulate metabolic hormones following SCI by triggering distinct cellular and metabolic pathways. While several ways in which enolase inhibition influences molecular cascades following SCI have been unearthed, the pathology of secondary injury in acute SCI remains complex, and additional research is needed to better understand the role of inflammatory mediators in these processes. Future investigation of ENOblock treatment should include its specific mechanism, neuroprotective effects, and involvement in modulating anti-inflammatory cytokines, chemokines, and growth factors. Overall, higher levels of NSE have been shown to have detrimental effects following acute SCI, and pending further investigation, ENOblock inhibition of enolase/NSE appears to be a promising therapeutic avenue for attenuating secondary damages of SCI.
